# Supplementary material for: Synthesis of Bioactive Silver Nanoparticles by a Pseudomonas Strain Associated with the Antarctic Psychrophilic Protozoon Euplotes focardii
Source: Mar Drugs. 2020 Jan 3;18(1):38. doi: 10.3390/md18010038 (PMC7024347; doi:10.3390/md18010038)
Supplement: Supplementary file 1 [file marinedrugs-18-00038-s001.pdf]

marine drugs

Supplementary files

Synthesis of Bioactive Silver Nanoparticles by a *Pseudomonas* Strain Associated with the Antarctic Psychrophilic Protozoon *Euplotes focardii*

Maria Sindhura John [1], Joseph Amruthraj Nagoth [1], Kesava Priyan Ramasamy [1], Alessio Mancini [1], Gabriele Giuli [2], Antonino Natalello [3], Patrizia Ballarini [1], Cristina Miceli [1] and Sandra Pucciarelli [1,*]

[1] School of Biosciences and Veterinary Medicine, University of Camerino, Camerino, 62032, Italy;sindhuramaria@gmail.com (M.S.J.); amruthjon@gmail.com (J.A.N.); kesavanlife@gmail.com (K.P.R.); alessio.mancini@unicam.it (A.M.); patrizia.ballarini@unicam.it (P.B.); cristina.miceli@unicam.it (C.M.)

[2] School of Sciences and Technology, University of Camerino, Camerino 62032, Italy; gabriele.giuli@unicam.it

[3] Department of Biotechnology and Biosciences, University of Milano-Bicocca, Milano 20126, Italy; antonino.natalello@unimib.it

* Correspondence: sandra.pucciarelli@unicam.it; Tel.: +39-0737-403231

Received: 30 November 2019; Accepted: 31 December 2019; Published: date

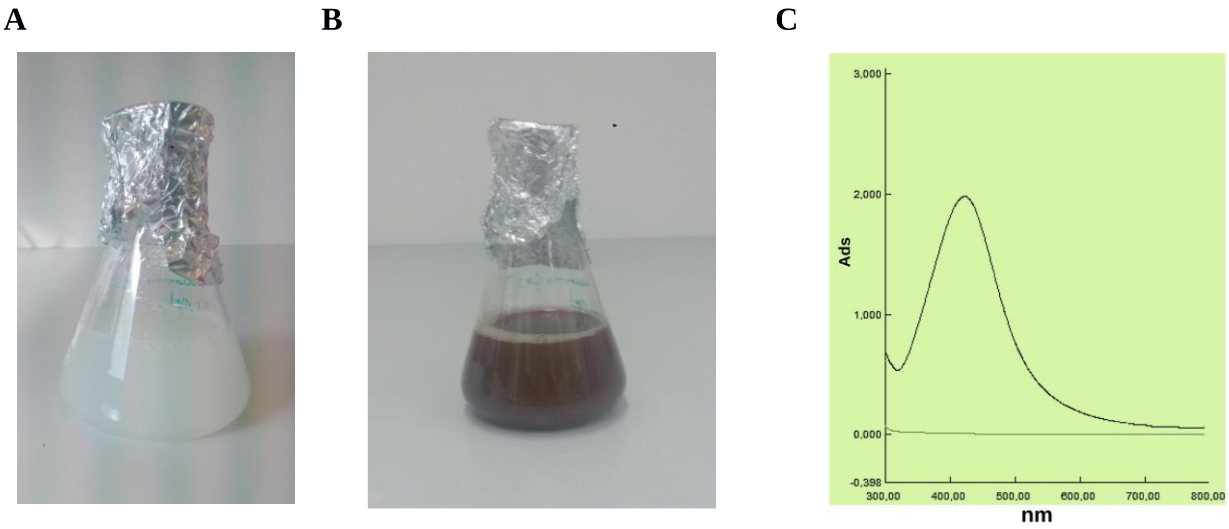

Figure S1: AgNP synthesis by *Pseudomonas sp* ef1. A and B: Visual observation of AgNP synthesis by medium colour change of a *Pseudomonas* sp ef1 culture from with (A) to dark brown (B) during 24 hrs of incubation with 1mM of $AgNO_3$. (C) UV–vis spectrum of *Pseudomonas* sp ef1 AgNPs. A small aliquot (0.1 ml) of the 24 h *Pseudomonas* sp ef1 culture was diluted with ddH2O and UV–visible spectra was recorded from 300 to 800 nm wavelength at room temperature.

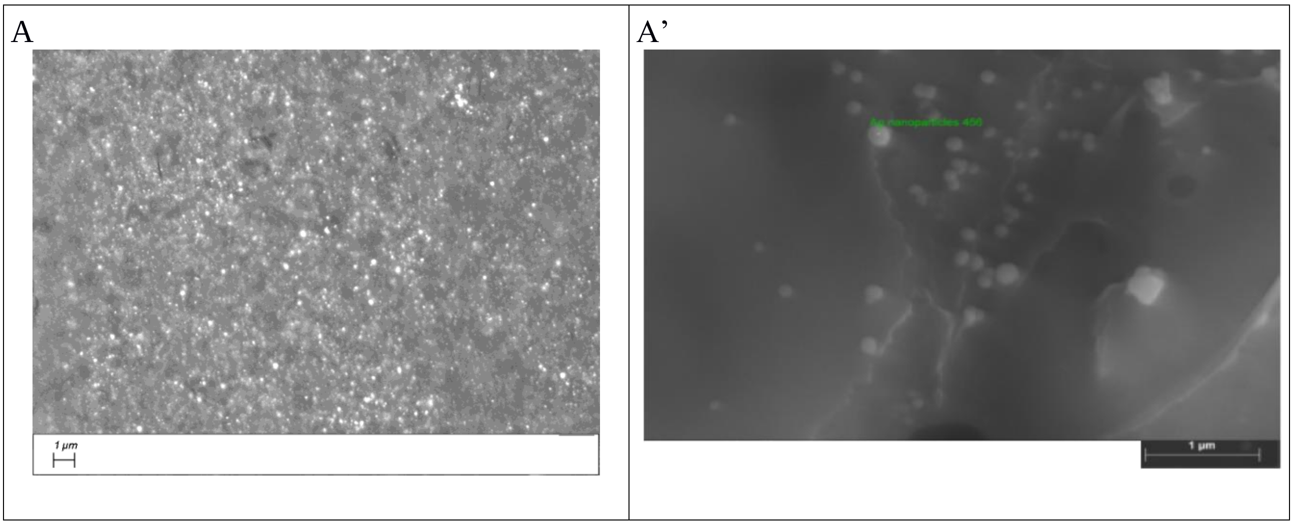

Figure 2. Scanning electron microscopic (SEM) images of the Pseudomonas sp ef1 AgNPs at different magnification. Bars: 1 μM.

Table S1. Antimicrobial activity of AgNPs synthesized by Pseudomonas *sp* against various pathogenic organisms. R* Resistance.

No	Microorganism	Bio-AgNps (A) (mm)	Chemical AgNps (B) (mm)	Increased zone size b/w A&B (A-B) (mm)	$AgNO_3$ (C) (mm)	Increased zone size b/w A&C (A-C) (mm)	Dist.Water (mm)
				Gram positive bacteria			
**1**	*Staphylococcus aureus*	15	12	3	9	6	R
**2**	*Staphylococcus epidermidis*	13	10	3	9	4	R
**3**	*Streptococcus agalactie*	13	10	3	8	5	R
				Gram negative bacteria			
**4**	*Escherichia coli*	17	14	3	10	7	R
**5**	*Klebsiella pneumoniae*	16	13	3	9	7	R
**6**	*Pseudomonas sp*	14	11	3	9	5	R
**7**	*Proteus mirabilis*	14	11	3	8	6	R
**8**	*Citrobacter koseri*	15	12	3	9	6	R
**9**	*Acinetobacter baumanii*	15	13	2	10	5	R
**10**	*Serratia marcescens*	14	11	3	8	6	R
				Fungi			
**11**	*Candida albicans*	15	12	3	7	8	R
**12**	*Candida parapsilosis*	12	10	2	8	4	R
